# Protein Corona Composition of Silica Nanoparticles in Complex Media: Nanoparticle Size does not Matter

**DOI:** 10.3390/nano10020240

**Published:** 2020-01-29

**Authors:** Laurent Marichal, Géraldine Klein, Jean Armengaud, Yves Boulard, Stéphane Chédin, Jean Labarre, Serge Pin, Jean-Philippe Renault, Jean-Christophe Aude

**Affiliations:** 1 Université Paris-Saclay, CEA, CNRS, Institute for Integrative Biology of the Cell (I2BC), 91198 Gif-sur-Yvette, France; geraldine.klein@u-bourgogne.fr (G.K.); yves.boulard@i2bc.paris-saclay.fr (Y.B.); stephane.chedin@cea.fr (S.C.); jean.labarre1@gmail.com (J.L.); 2 Université Paris-Saclay, CEA, CNRS, NIMBE, Laboratoire Interdisciplinaire sur l’Organisation Nanométrique et Supramoléculaire, 91191 Gif-sur-Yvette, France; serge.pin@cea.fr (S.P.); jean-philippe.renault@cea.fr (J.-P.R.); 3 UMR Procédés Alimentaires et Microbiologiques, Equipe VAlMiS (Vin, Aliment, Microbiologie, Stress), Institut Universitaire de la Vigne et du Vin, AgroSup Dijon, Université de Bourgogne Franche-Comté, rue Claude Ladrey, BP 27877, 21000 Dijon, France; 4 Laboratoire Innovations technologiques pour la Détection et le Diagnostic (Li2D), Service de Pharmacologie et Immunoanalyse (SPI), CEA, INRA, 30207 Bagnols-sur-Cèze, France; jean.armengaud@cea.fr

**Keywords:** silica nanoparticles, protein corona, curvature effect, high-throughput proteomics, Bayesian statistical analysis

## Abstract

Biomolecules, and particularly proteins, bind on nanoparticle (NP) surfaces to form the so-called protein corona. It is accepted that the corona drives the biological distribution and toxicity of NPs. Here, the corona composition and structure were studied using silica nanoparticles (SiNPs) of different sizes interacting with soluble yeast protein extracts. Adsorption isotherms showed that the amount of adsorbed proteins varied greatly upon NP size with large NPs having more adsorbed proteins per surface unit. The protein corona composition was studied using a large-scale label-free proteomic approach, combined with statistical and regression analyses. Most of the proteins adsorbed on the NPs were the same, regardless of the size of the NPs. To go beyond, the protein physicochemical parameters relevant for the adsorption were studied: electrostatic interactions and disordered regions are the main driving forces for the adsorption on SiNPs but polypeptide sequence length seems to be an important factor as well. This article demonstrates that curvature effects exhibited using model proteins are not determining factors for the corona composition on SiNPs, when dealing with complex biological media.

## 1. Introduction

Nanoparticles (NPs) are being used in all kinds of everyday consumer products such as food, cosmetics, or medicine [1]. Even though those NPs are deemed safe by many national authorities, their ability to be internalized [2,3] and translocated from an organ to another [4,5] raises concern about potential harmful effects. The potential toxicity of a given NP cannot be well monitored by classical toxicological studies [6], and new approaches have to be developed [7,8]. Indeed, NPs’ presence in a biological medium can trigger indirect biological effects (e.g., cytotoxicity [9], activation of inflammatory responses [10]). It is now established that the main reason for these pernicious effects comes from the capacity of NPs to interact with biomolecules, mainly proteins, found in their surroundings. These latter form a so-called “protein corona” on NPs’ surfaces [11]. The composition of the corona was shown to have an impact on NPs’ biodistribution [12], susceptibility to biodegradation [13], cell internalization mechanism [14], and the immune response they trigger [15]. Consequently, the composition of this corona can lead to detrimental effects regarding the target specificity of nano-carriers used in nanomedicine [16]. Conversely, it could be used as a strategy to develop NPs based therapeutic approach targeting specific cellular pathways [17].

Factors ruling the protein corona formation have been studied for a long time [18]. The environment properties (e.g., pH, temperature, ionic strength, shear stress [19,20,21]) can have a large influence on the final corona composition and structure. However, many determining factors lie in the intrinsic properties of NPs and proteins, especially those related to their surface physiochemistry such as charge, hydrophobicity, shape, and size [18,22]. In particular, the NP size factor has been shown to play a substantial role in the protein corona composition [22,23,24], but no detailed explanation was given about the fundamental mechanisms involved. One hypothesis is that smaller spherical NPs (diameter < 10 nm) have a very high curvature while larger ones (diameter > 100 nm) can be seen as a flat surface relative to protein sizes, the typical diameter of monomeric globular proteins being around 3 to 6 nm. Indeed, this curvature difference can affect greatly the structural fate of adsorbed proteins. The general assumption is that the more the surface of an NP is curved, the more the structure of an adsorbed protein is preserved. This was demonstrated with model proteins such as lysozyme [25,26], bovine serum albumin [27], human carbonic anhydrase I [28], blood coagulation factor XII [29], or cytochrome C [30]. However, there are exceptions where a curved surface can be more damaging for protein structure than a flat one [31,32]. Damages regarding protein structure integrity have obvious consequences in terms of biological function, but can also lead to different protein corona compositions and structures [33], and may be part of the actual cause of the NP size effect seen previously. Besides, to our knowledge, the protein size factor has not been directly studied so far. Yet, some results indicated a possible influence of this factor in protein-NPs interactions [27,32].

The majority of the previous studies used model proteins, usually well structured, but not always relevant regarding the actual protein corona formed in biological media. To overcome this limitation, proteomic studies were performed on protein coronas formed in complex media [24,33,34,35,36,37,38]. Some of them even directly addressed the influence of the NP size [24,36] but, even though protein corona composition differences were established, no general explanations were proposed about the physicochemical basis underlying those differences. The main limitation of those studies came from the biological media used (i.e., blood fractions) which present very high dynamic range in terms of protein abundance [39] limiting the analyses to the most abundant proteins [24]. To avoid these limitations, an analysis based on brewer’s yeast protein extracts was proposed [34]. Using this approach, it was found that proteins containing disordered regions were largely overrepresented in protein coronas formed around silica nanoparticles (SiNPs) [33], and that these regions were in direct contact with the silica surface [40]. These studies were done on small aggregated SiNPs (without any flat surface). Whether this could be generalised to SiNPs of any size, to any negatively charged NP and whether the high affinity of disordered proteins was curvature-specific remained to be determined.

One specific aspect that was also lacking in these proteomic studies was the correlation between the NP size and the molecular weight of the adsorbed proteins. For instance, regarding synthetic polymers adsorbed on particles, it is known that large polymers adsorb more readily than small ones [41]. However, this effect seems independent of the particle curvature as it is similar to the one observed on flat surfaces where larger proteins [42] or polymers [43], having more contact points with the surface, are more affine. Nevertheless, a specific effect of the curvature has been proposed for polymers [44] and large proteins [45], with high curvature limiting their adsorption by steric effects.

The aim of this work was to investigate both the curvature effects of NPs and the physicochemical properties of the proteins of the corona formed on the surface of SiNPs. We analysed the protein coronas formed on SiNPs synthesised by the same process but with different sizes (8.3, 33.0, and 78.0 nm of diameters denoted S10, S30, and S80 respectively hereafter) to study the consequences on protein adsorption at the physicochemical level. Then, we used the same set of SiNPs with a complete yeast protein extract, containing most of the yeast globular and hydrophilic proteins in natural abundances. A label-free proteomics approach and statistical modelling methods were used to characterize the protein corona composition [34] and the physicochemical properties of the proteins that drive the corona formation. Our approach aims at providing an archetypal example of adsorption in competitive conditions.

## 2. Materials and Methods 

### 2.1. Nanoparticle Characterization

Silica nanoparticles (SiNPs) were custom made by Corpuscular Inc. (Cold Spring, NY, USA) in order to have monodisperse nanospheres of different diameters. Their synthesis is adapted from the Stöber method [46]. Three samples of different diameters were purchased and stored in pure water. Before use, SiNPs were thoroughly characterised. Mass concentration was measured by desiccation and dry mass weighing. Size, shape, and dispersity were assessed by small-angle X-ray scattering (SAXS) using a Xeuss 2.0 spectrometer (Xenocs, Grenoble, France) located at the CEA Saclay/IRAMIS/NIMBE (France). Data fitting was done using a sphere model from the SASview software [47]. Detailed information on the use of SAXS technique for nanoparticle research can be found here [48]. Zeta (ζ) potential was measured by electrophoretic light scattering performed on a Zetasizer Nano-ZS (Malvern Instruments, Malvern, UK). Measurements were performed in triplicate.

### 2.2. Sample Preparation and Adsorption Isotherms

Yeast protein extracts were prepared from the *Saccharomyces cerevisiae* strain S288C (*Mata SUC2 mal mel gal2 CUP1*) [49] as previously described [33,34]. ^35^S-labelled proteins from the same strain were also produced using the method described in [34]. Equilibrium adsorption isotherms were performed by a depletion method (amount of adsorbed proteins obtained by subtracting the total protein fraction with the non-adsorbed protein fraction). A fixed concentration of SiNPs (1 g·L^−1^) was mixed with varying concentrations of yeast protein extract (mix of ^35^S-labelled and unlabelled protein extracts) ranging from 0 to 3 g·L^−1^ and diluted in Dulbecco’s phosphate-buffered saline (DPBS) at pH 7.4 [50]. After 3 h of gentle mixing at 4 °C, samples were centrifuged (20,000× *g*, 10 min), and the supernatants radioactivity was measured by liquid scintillation (Wallac 1409 DSA liquid scintillation counter). Isotherms were fitted using a Langmuir-Freundlich model [51] (Equation (1)).

(1) $$m_{ads}=\frac{m_{\infty }\cdot K_{ads}\cdot C^{n}}{1+K_{ads}\cdot C^{n}},$$

where *m_ads_* (mg·m^−2^) is the amount of adsorbed protein (expressed in surface area in order to take into account the SiNP surface specificity differences) and *C* (g·L^−1^) is the concentration of non-adsorbed proteins. The deduced constants are *m_∞_* (mg·m^−2^) the maximum amount of adsorbed protein, *K_ads_* (L·g^−1^) the adsorption constant (proportional to the affinity), and *n* the heterogeneity index which accounts for the surface heterogeneity as well as multilayer adsorption mechanisms (characteristic of the Freundlich model) [52].

### 2.3. Label-Free Shotgun Proteomics

Samples (analyses were performed in triplicates) used for mass spectrometry analyses were obtained by mixing SiNPs (final concentration of 1 g·L^−1^) and protein extract (2.70 g·L^−1^ for S10, 1.25 g·L^−1^ for S30, and 0.60 g·L^−1^ for S80) diluted in DPBS. Protein concentrations were chosen in order to be in excess of proteins according to the adsorption model (see Section 3.1) compared to the available SiNP surface with 15–17% of proteins actually adsorbed. Then, after a 3 h incubation time, adsorbed proteins were centrifuged (20,000× *g*, 10 min), washed with DPBS, and finally resuspended in DPBS containing 1% sodium dodecyl sulfate (SDS). Then, a total of 9 µg of adsorbed proteins were diluted with lithium dodecyl sulfate (LDS) 3X (Invitrogen) to obtain 30 µL of samples, or eventually precipitated with trichloroacetic acid 10% final, centrifuged, and diluted into 30 µL of LDS 1X. The 30 µL samples were subjected to a short NuPAGE 4–12% (Invitrogen) SDS-PAGE migration and for each sample the whole proteome was excised as a single polyacrylamide band as previously recommended [53]. Then, samples were prepared as described in [33] for protein proteolysis and the resulting peptides were analysed by nanoLC-MS/MS by means of a Q-Exactive HF tandem mass spectrometer (ThermoFisher, Waltham, MA, USA) coupled to an UltiMate 3000 LC system (Dionex, Sunnyvale, CA, USA), and operated as previously described [33]. MS/MS spectra were searched using MASCOT 2.2.04 software (Matrix Science, Boston, MA, USA) against the SGD yeast database (5885 sequences). Every peptide matching with a MASCOT peptide score below a *p*-value of 0.01 was filtered and assigned to a protein according to the principle of parsimony. A protein was considered to be validated when at least two different peptide sequences were detected and was assigned a spectral count [54] value defined as the number of spectral copies of detected peptides. Relative protein enrichment between samples recovered after adsorption and the initial mixture was estimated using the Log Fold-Change (LFC). For each detected protein, the LFC is calculated as the Log_2_ of the spectral count ratio (in each condition and average over triplicates) as described in [33] (see ShotgunData in Supplementary Materials). A negative binomial model has been used to define the subsets of highly adsorbed proteins (HAP) using the same thresholds as reported in [33]: (i) a LFC greater than one (i.e., a fold-change greater than 2); (ii) an adjusted *p*-value inferior to 0.05.

### 2.4. Polypeptide Sequence Statistical Analysis

All datasets were processed and analysed using the R language [55]. Amino-acids primary sequences were retrieved from the UniprotKB database release 2018_06. Protein features such as sequence length (SeqLen), as the number of AA in the canonical sequence, percentage of Arg (%Arg), and percentage of positive charges (%PosAA), as the sum of Arg and Lys relative to SeqLen, were calculated from the annotated polypeptide sequences. Protein disordered regions were retrieved from the meta-heuristic web-server PONDR (predictor of naturally disordered regions, Molecular Kinetics Inc., Indianapolis, IN, USA). Highly structured regions of proteins, as defined in the hydrophobic cluster analysis (HCA) method [56], were calculated using the Seg-HCA software [57]. Both disordered and highly structured regions of proteins were converted to percentages (respectively named %DisReg and %HyClus) of the full sequence lengths in subsequent analysis.

Statistical differences between pairs of features were computed using the Kolmogorov–Smirnov test for distribution comparisons (including a bootstrap procedure to handle discrete features such as sequence lengths [58,59]), and the Wilcoxon rank sum test for distribution location comparisons. Resulting *p*-values were adjusted for multiple comparisons by controlling the false discovery rate using the Benjamini–Hochberg method [60]. Comparisons between the three NP sizes were performed using a Welch’s one-way ANOVA. This frequentist approach was complemented using a Bayesian framework (as implemented in the BayesFactor R package [61]). Bayesian Student *t*-tests [51] were used to assess the log odd-ratios of feature distribution differences. Bayesian factor analysis was also used for linear regression model calculation, selection, and comparison.

## 3. Results

### 3.1. Adsorption Models

**Figure 1:**
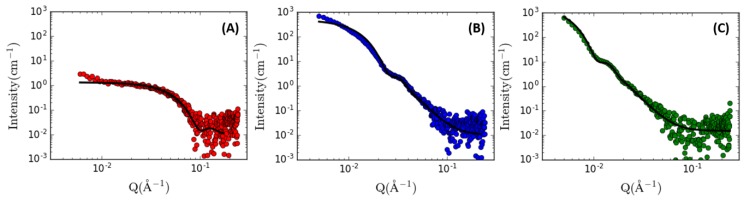
Small-angle X-ray scattering curves of silica nanoparticles in pure water: (**A**) S10; (**B**) S30; and (**C**) S80. Fitting of the experimental data (colored dots) by a sphere model (black curves) using SASview software.

Nanoparticle geometry was characterised using small-angle X-ray scattering (SAXS). This in situ technique was preferred to transmission electron microscopy as it can be performed in solution and is not susceptible to sampling biases. SAXS can give information related to the structure of the nanometric objects suspended in solution. Here, scattering curves of the S10, S30, and S80 SiNPs samples (Figure 1) showed that the objects were spherical (typical oscillation pattern), monodisperse (pronounced oscillations), and with respective diameters of 8.3, 33.0, and 78.0 nm (Table 1), as measured using a spherical model (size is inversely proportional to the first oscillation position on the *x*-axis).

The curvature was deduced from those diameters and showed large differences between NPs, with an order of magnitude of difference between S10 and S80. Their surface charges were also assessed: S10 is almost neutral (slightly negatively charged) while S30 and S80 are highly negatively charged at pH 7 (Table 1). This reduced acidity for smaller SiNPs is known and is due to the smaller amount of ionizable silanol groups found at the silica surface [62]. So, S10 was clearly distinct, both in terms of curvature and surface charge compared to the other SiNPs.

**Figure 2:**
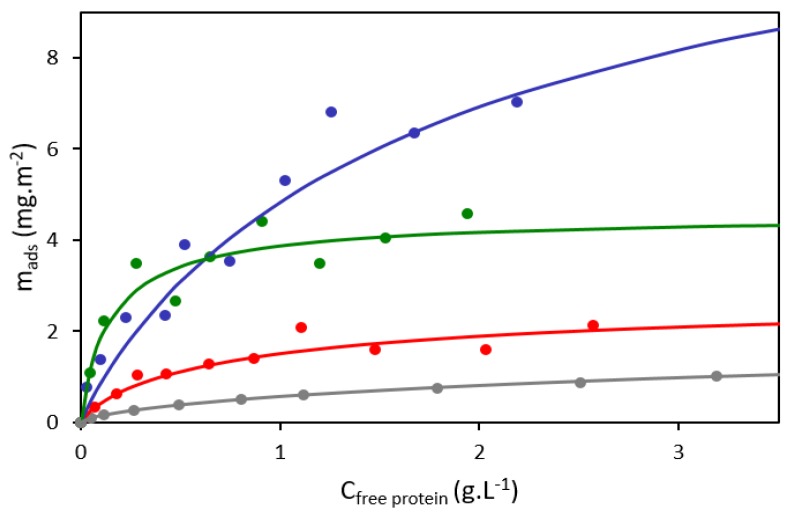
Adsorption isotherms of yeast protein extract (S288c) adsorbed on different silica nanoparticles in Dulbecco’s phosphate-buffered saline (DPBS) buffer (pH 7.4). The curves depict isotherms fitted by the Langmuir–Freundlich model for the following silica nanoparticles: S10 (**red**); S30 (**blue**); S80 (**green**); and polydisperse nanoparticles (NPs) (**grey**) taken from Mathé et al. [34]. The dots are experimental points associated to a single isotherm.

In a first step we analysed the adsorption of a radioactively labelled cell extract on S10, S30, S80, and on a polydisperse silica sample studied previously [34]. Contrary to isotherms obtained with model proteins [63,64], isotherms depicted on Figure 2 did not possess a well-defined plateau indicating the saturation of the surface. 

Consequently, the classical Langmuir adsorption model could not be applied. The hybrid Langmuir–Freundlich model (Equation (1), see Section 2.2) seemed more suited as it can take into account surface heterogeneity and multilayer adsorption [51,52]. Thus, experimental measurements were fitted to this model and summarised in Table 2. Both *m_∞_* (maximum amount of adsorbed protein) and *K_ads_* (adsorption constant) changed substantially between the systems. In particular, the large SiNPs (S30 and S80) had larger *m_∞_* than their smaller (S10) or polydisperse counterparts, resulting in more proteins being able to adsorb on a given surface. Similar observations were made with gold NPs [65] for which the protein corona thickness was found to be proportional to the NP size. The explanation given was that small NPs form incomplete coronas (due to steric repulsion of proteins on the curved surface) while large NPs form multilayered coronas [66]. However, the *m_∞_* of S80 was between the *m_∞_* of S10 and the *m_∞_* of S30 indicating that other factors may also be at play. *K_ads_* varied also greatly with more than an order of magnitude of difference between the least and the most affine SiNPs, but no tendency could be observed in relation to the SiNP size or dispersity. As expected, the heterogeneity index *n* was also very distinct between monodisperse and polydisperse SiNPs. The former had a *n* slightly smaller than one while the latter had a *n* of 0.69. This confirmed the aggregated state of the polydisperse SiNPs. *n* strictly inferior to one can also indicate the formation of multilayers of adsorbed protein [52] which is likely to happen due to the presence of non-directly bound proteins found in coronas [37].

### 3.2. Analysis of the Highly Adsorbed Protein (HAP) Subsets

**Figure 3:**
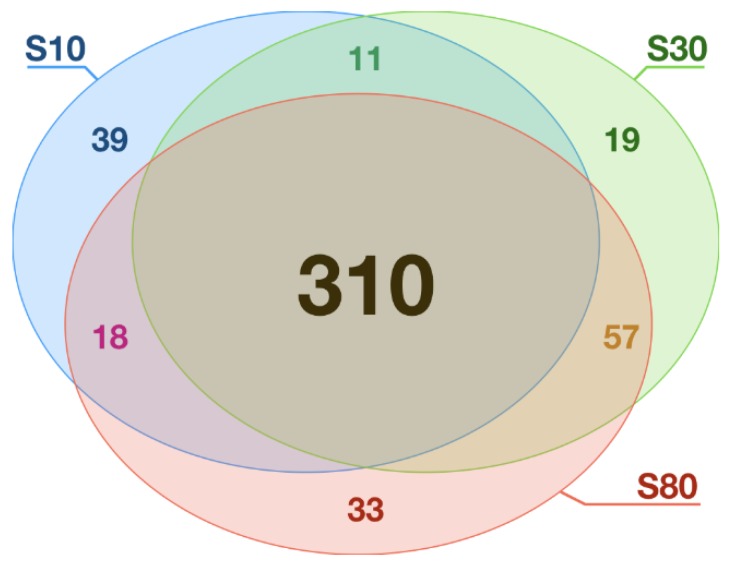
Venn diagram of the highly adsorbed proteins (HAP) on the three silica nanoparticles. This diagram depicts the number of shared highly adsorbed proteins, in all possible overlapping sets, on the three silica nanoparticle S10, S30, and S80.

Considering these differences in terms of protein adsorption, we conducted an extensive proteomic analysis to identify the proteins associated to different SiNPs. A total of 33,683 peptide sequences were identified by tandem mass spectrometry and 2515 polypeptides were confidently monitored with at least two peptides in the whole dataset. Proteins found in protein coronas can be divided into three categories: proteins that are as abundant in the corona as in the initial yeast protein extract, proteins that are less abundant in the corona, and proteins that are more abundant. From this last category, the proteins that are the most enriched in the corona form the highly adsorbed protein (HAP) group (see Section 2.3). For the S10/S30/S80 SiNPs, 378/397/418 HAP were identified out of 2369/2390/2423 total proteins detected in the nanoLC-MS/MS shotgun analysis. The overlaps between the HAP subsets at each NP size is shown in Figure 3. The large majority of HAP (64%, 310 out of 487 unique protein IDs) were common between the three NP sizes, 17% were found in two subsets, and less than 19% were specific to a single NP size. Interestingly, no HAP subset seemed to be more specific than the others.

For every HAP primary sequence, using the same analysis as performed in [33], protein features, corresponding to physicochemical properties relevant for the adsorption (such as the percentage of positively charged AA) were calculated (see Section 2.4). To estimate if the slight differences of HAP for each NP size induced changes in proteins features, a Welch’s one-way ANOVA for each feature was performed (Figure S1): no significant difference between the HAP subsets of each NP size could be found (all adjusted *p*-values were inferior to 0.001). Associated Bayes factor (BF) log odd-ratios, log_e_(BF_01_) in favour of the null hypothesis, were in the range of 1.98 to 5.26, and corroborated the absence of differences between the features of HAP subsets for each NP size. 

We then compared the distributions of protein features of the HAP subsets at each NP size versus all proteins detected by the shotgun analysis in the yeast extracts. One-sided two-samples Kolmogorov–Smirnov tests (Table S1) showed that, for every feature, the cumulative distribution function of the HAP subsets and set of all detected proteins were significantly different. Moreover, the one-sided Wilcoxon rank sum tests (Table S1) also showed that the distribution location was shifted between the HAP and detected protein sets. Both tests showed that regardless of the NP size, HAP subsets have specific features in comparison to the set of all detected proteins. This had previously been shown with coronas formed on polydisperse SiNPs [34]. Indeed, we showed that highly adsorbed proteins were: (i) enriched in disordered regions; (ii) conversely depleted in structured regions; and (iii) enriched in positively charged AA (particularly Arg). The present statistical analysis confirmed all these conclusions and extended it for all sizes of monodisperse SiNPs. Besides, the size of the polypeptides was also compared, and we observed that longer primary sequences were more prone to adsorption.

### 3.3. Quantitative Analysis of Protein Physicochemical Properties

Previous analyses were conducted using HAP subsets irrespective of the level of adsorption on the NPs surface. While these analyses can highlight the main properties of the adsorbed proteins, a quantitative approach using the log fold-change (LFC) as a proxy for the level of adsorption [67] was also performed. Table 3 summarises the correlation coefficient calculations between the LFC and each protein feature of this study. To assess the significance of these correlations, a test for association, using the Pearson’s product moment correlation coefficient (PPMCC), was performed for each paired sample. All adjusted *p*-values (see Section 2.4) were inferior to 10^−3^, showing that correlation coefficients were significant. Moreover, associated Bayes factors log odd-ratios were negative (Table 3), supporting the alternative hypothesis H_1_ (i.e., the correlation coefficient ***ρ*** is not equal to 0); in addition, all values were in favour of a strong (from −10 to −30) or very strong (from −30 to −100) evidence for H_1_ according to [68]. Table 3 also shows that both S30 and S80 NPs had similar correlation profiles while S10 NP correlation profile was markedly different with a higher percentage of disorder regions coefficient ***ρ***. The correlation coefficient ***ρ*** can be interpreted as representing an effect size which measures the magnitude of a phenomenon, here the strength of the correlation between the protein features and the LFC. We observed small to moderate effect size (as in Gignac and Szodorai’s guidelines [69]) for some protein features. Two have small (0.1–0.2) effect size: the percentage of charged AA (%PosAA), and the percentage of hydrophobic clusters (%HyClus). Three have moderate (0.2–0.3) effect size: the sequence length (Seqlen), the percentage of Arg (%Arg), and the percentage of disordered regions (%DisReg). The effect size for %DisReg in S10 NPs could be described as large according to [69]. Overall, these results showed that several features contributed to the adsorption, and we set out to characterise the contribution of combinations of features.

To identify potential links between the LFC and the physicochemical features, we fitted linear regression models using the LFC as a response variable and the physicochemical properties as covariates, for each NP size (for predictive models see Findlay et al. [70] for example). Using a Bayesian factor analysis against all possible models (see Section 2.4) we observed that the best fitted model was the same for each NP size:

(2) $$LFC_{i}=\beta_{0}+\beta_{1}\cdot SeqLen_{i}+\beta_{2}\cdot \%DisReg_{i}+\beta_{3}\cdot \%Arg_{i}+\epsilon_{i},$$

where *β*_0_, *β*_1_, *β*_2_ and *β*_3_ are the regression parameters and ε the error variable (Table S2). Extending this model with the percentage of positively charged AA and/or the percentage of AA in hydrophobic clusters did not improve the model (Table S3). Hence, regardless of NP size, the best fitted model needed the same three covariates (SeqLen, %DisReg and %Arg). To identify the contribution of individual features, we used top-down/bottom-up approaches where each covariate was omitted/added, one at a time (Table S4). We observed that in all cases but one, the protein sequence length is the most influential variable explaining the LFC variations. The only exception is given by the bottom-up analysis for the smallest NPs S10 where the percentage of AA in disordered regions is the most influential variable closely followed by the protein sequence length (Table S4d).

**Figure 4:**
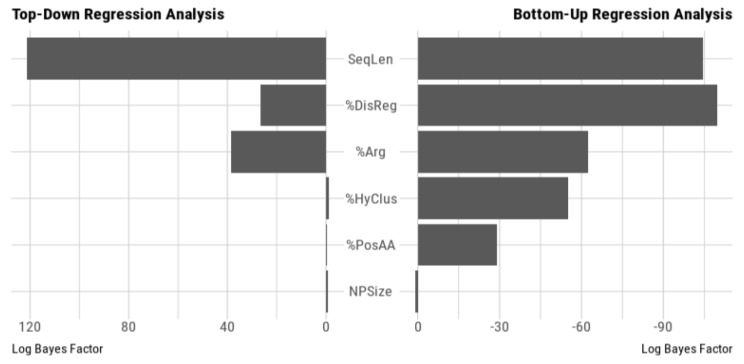
Top-down/bottom-up Bayes factor regression analysis. The left bar chart depicts the changes of the Bayes Factor (*bf*), as *log_10_(1/bf)*, when variables (indicated in between both charts) are iteratively omitted in the linear regression model. The right chart depicts the changes of the Bayes Factor when variables are iteratively added to the linear regression model. Variables are respectively: the sequence length (*SeqLen*); the percentage of AA in disordered regions *(%DisReg*); the percentage of Arg AA *(%Arg*); the percentage of AA in hydrophobic clusters (*%HyClus*); the percentage of positively charged AA (*%PosAA*); the size of the NP (*NPSize*).

To estimate the contribution of the NP size, we used a similar approach and built a global model by gathering all available data (see Section 2.4) and including NP size as a new covariate. The Bayesian factor analysis against all possible models showed that the previous, three covariates model (Equation (2)) remained the best fitted to explain the correlations between the LFC and the protein physicochemical properties. Moreover, the top-down/bottom-up approaches revealed that the NP size is the least influential covariate to explain the LFC variations as shown in Figure 4.

## 4. Discussion

Adsorption isotherms showed that large SiNPs could adsorb more proteins per area unit than small (monodisperse or polydisperse) SiNPs. The comparison with the study done on gold NPs [65], in which the authors showed that the protein corona thickness is correlated with the NP size, is tempting. However, one fundamental factor needs to be highlighted: aggregation of the NPs. Indeed, gold NPs mixed with proteins did not undergo any noticeable aggregation while SiNPs tend to aggregate when mixed with proteins [64]. So, in this study, an additional explanation can be proposed. Due to the differences of surface charges between small and large SiNPs, the former may aggregate faster [66]. This could lead to aggregate formation before all the surface is covered by proteins. Therefore, the NP surface actually available for protein adsorption may be smaller than estimated. Consequently, the different amounts of adsorbed protein on SiNPs with different sizes could also be due to a surface charge effect rather than solely a direct curvature effect. Kinetic studies of the aggregation process during the protein corona formation would be necessary to assess this hypothesis. 

We have completed this study by a proteomic shotgun analysis of the corona composition of each SiNP size. The statistical analysis of the highly adsorbed proteins subsets showed clearly that there are no significant differences among the three SiNP sizes (Figure 3). Interestingly, we observed the same phenomenon for the most non-adsorbed proteins (Figure S2). To evaluate if the absence of size effect for the protein corona composition was still valid at even lower curvature, we incubated yeast protein extracts with silica (quartz) microparticles (diameters in the range of [0.5–10] µm) and microfibers (diameters in the range of [1–2] µm). At this scale, high-throughput proteomic methods become intractable due to the low specific surface (i.e., a lower surface area per unit of mass). Thus, we used a combination of fluorescent microscopy and GFP tagging [40] to follow the fate of selected proteins previously identified by shotgun analysis (GFP on its own does not adsorb on silica surfaces [71]). The supporting material Figure S3 shows that the tested proteins maintain their adsorption/non-adsorption behaviour on both NPs and microparticles. Additionally, one can notice that the adsorption, when it occurs, is inhomogeneous between the objects (Figure S3A,B). Thus, there is an intrinsic diversity in silica surfaces that influence the corona composition at a single object level, much more than the intrinsic curvature of the object. Nevertheless, properties of the proteins specific of only one NP size (Figure 3) can be different and possibly be responsible for functional modifications within cells. Thus, using protein features that were previously shown to be relevant for the adsorption (i.e., percentages of disordered and structured regions, Arg AA, and positively charged AA), as shown in Klein et al. [33], we compared these distributions for each NP size. Our results showed that these features were relevant, regardless the NP size and small differences in the HAP subsets have no impact on the overall protein physicochemical properties describing protein adsorption. This suggests, at least from a qualitative point of view and considering the proteins that are the most impacted by the presence of SiNPs, that the size of the NPs does not affect the adsorption process. We also conducted a quantitative study of the adsorption process for the whole set of proteins (including the HAP) using a statistical modelling approach to analyse the correlations between the LFC and the protein features. We showed that the LFC could be used as an effective approximation of the adsorption level as it quantitatively measures the ratio between the number of adsorbed proteins on the NP surface and the number of proteins in the cellular extract, as revealed by the proteomic analyses. Our results showed that correlations are statistically significant for all features and effect sizes are in the range of small to moderate. Still, we observed a slight increase for the percentage of disordered/structured regions for the smallest SiNP: the rigidity of the protein might be a limiting factor for adsorption owing to the high curvature of the surface [65]. Nevertheless, we have to highlight the existence of biases in the selected features, due to collinearity in their definitions (e.g., the calculation of the percentage of Arg and positively charged AA both involved Arg). To account for this effect, the best linear model using Bayes factors was computed as a selection criterion. This model is the same for every NP size, though different coefficients were applied (Table S2). The three datasets were also gathered in order to compute a global model with the size of the NPs as a new covariate. Using the same approach, completed with top-down and bottom-up analyses, we showed that the size of the SiNPs is not a relevant parameter to explain the protein adsorption of a protein extract. Finally, in this study, we showed that the curvature of the SiNPs has no consequences on the protein corona composition but that it impacts the maximum amount of adsorbed proteins.

Regression models provide insights on the links between protein features involved in the adsorption mechanism. Interestingly, two features could be discarded: (i) the percentage of hydrophobic clusters, which is negatively correlated with the percentage of disordered regions; (ii) the percentage of positively charged AA, which is colinear with the percentage of Arg. For the first point, the overlap between both criteria were already mentioned in the literature [57]. Still, the regression models showed that disordered regions account to a larger extent than the more structured parts of the proteins. The second point highlights that, among positive charges, which play a crucial role in the interaction with the negatively charged surface of the SiNPs, Arg is the most prone to initiate this interaction as it was already described [31,60]. It is noteworthy that among the protein features analysed we also included the length of the protein primary sequence as a new parameter as we thought it may also play a role in adsorption [42]. Using HAP subsets, we showed that longer polypeptides are more prone to adsorption (Table S1). This result was confirmed by correlation analysis with the LFC using the whole set of proteins (Table 3). We also observed that using Bayes factor analysis the protein sequence length has been selected as a covariate for the best fitted model (Equation (2)) regardless of the SiNP size. Moreover, the F-test statistics (Table S3) associated with the sequence length is the highest in each model (S10, S30, and S80) indicating that this feature is the most influential parameter among the covariates. We can also comment the role of protein sequence length in adsorption. This effect is well known for polymers [43], and can be explained by the fact that larger (bio)polymers have also a larger number of putative anchoring points, and thus higher affinity constants. Indeed, this larger number of anchoring points makes the adsorption almost irreversible and larger (bio)polymers progressively replace smaller ones that adsorb, but also desorb, more quickly [72,73].

**Figure 5:**
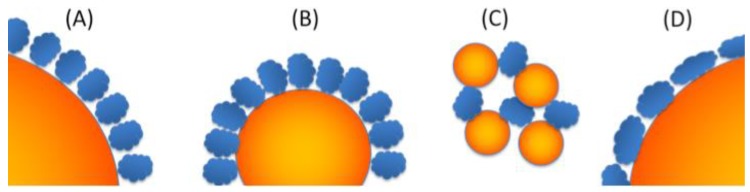
Schematic representation of proteins adsorbed on curved surfaces. Proteins (in blue) adsorbed on a NP (in orange) with low curvature (**A**), high curvature (**B**) in a compact way. Protein-NP interactions leading to a closed network (**C**). Proteins adsorbed on a large NP in a non-optimal way (**D**).

The combination of the isotherm and proteomic data gives an unusual picture of protein adsorption. Depending on the surface curvatures, the same protein adsorbs, in almost the same proportion, but the overall corona thicknesses are different. A possible explanation for this curvature effect would be a steric control. For low curvature (large particles, Figure 5A) a steric hindrance appears between neighbouring proteins that does not allow the entire available surface to be accessible. Increasing the curvature allows to optimize the packing on the surface (Figure 5B), but another type of steric hindrance can be identified for very small particles. When the size of the NPs is in the range of the size of the proteins, the formation of protein nanoparticle aggregates, recently observed by neutron scattering [64,74] leads to the formation of a porous network that renders a significant proportion of the NP surfaces inaccessible (Figure 5C). We must notice that such an optimal ratio of 10 between local curvature and polymer size was already identified in synthetic polymer adsorption on rough surfaces [44,75]. A complementary explanation could be linked to a change in orientation between S30 and S80 (Figure 5B,D). This change was observed for purified proteins and was associated with a stronger interaction of the proteins with the surface when the curvature decreases [26], which leads to protein lying flatter and more destructured on the surface. Such a phenomenon (less interaction leading to a thicker adsorption layer) was also described for synthetic polymers since the pioneering work of De Gennes [43]. Indeed, the affinity constants measured (Table 2) were lower for S30 than for S80. In our case, the origin of the lower affinity constant on S30 compared to S80 could be that the curvature induces a lower structuration of water on its surface which in turn decreases the entropic contribution due to water desorption upon protein adsorption. This model could even explain the specific Langmuir–Freundlich behaviour of S30, as the protein having less interaction with the surface may have more interaction with other partners, thus more easily forming multilayers. We can hypothesize that, despite their identical composition, the interactome [37] of these coronas may be different in their internal organization, a point to be verified in the future.

## 5. Conclusions

Most of the proteins (64%) preferentially adsorbed on silica surfaces are common to the three NPs, whatever the size of the NPs. Thus, the size factor appears to be less determinant for protein corona composition than previously reported [24,36]. Recently, the importance of an ever-increasing number of determinant factors for the protein corona has been questioned [76]. It appeared that even very important factors such as surface chemical groups may not be as determinant as what one could assume. When interpreting proteomic analyses, one pitfall that needs to be avoided is to keep seeking for an effect while there may not be. This could lead to biased results and exaggeration of phenomena.

Nevertheless, the 19% specific proteins, which adsorb only on one particular size of SiNPs, may be important for the biological impact of the NPs. Still, we are far from being able to understand why or even if these few proteins interact specifically with NP of certain size when surrounded with thousands of putative protein partners.

The structural determinants associated to this pro-adsorption behaviour are the one already identified on silica of different origins, and shapes namely: (i) enrichment in arginine; (ii) enrichment in disordered regions. Arginine residues are obviously involved in the electrostatic interactions driving the adsorption whereas the destructuration allows the protein to maximize its interaction with the surface at a minimal structural cost. An original observation is here the role of protein length. Larger proteins adsorb more readily, regardless of the particle size.

## Figures and Tables

**Table 1:** Physical diameter and ζ potential of the silica nanoparticles. Measurements done by small-angle X-ray scattering (in pure water) and electrophoretic light scattering (in phosphate buffer 0.1 mol·L^−1^, pH 7), respectively.

	Physical Diameter (nm)	Curvature (nm^−1^)	ζ Potential (mV)
**S10**	8.3 ± 1.2	0.230	−4.6 ± 0.8
**S30**	33.0 ± 4.3	0.061	−20.1 ± 1.0
**S80**	78.0 ± 8.6	0.026	−22.9 ± 0.9

**Table 2:** Fittings of the adsorption isotherms by the Langmuir–Freundlich adsorption model. This model provides the maximum amount of adsorbed protein (*m_∞_*), the adsorption constant (*K_ads_*), and the heterogeneity index (*n*) [51]. Polydisperse SiNP data come from Mathé et al. [34] and are given for comparison purpose.

	*m_∞_* (mg·m^−^²)	*K_ads_* (L·g^−1^)	*n*
**S10**	2.9	1.1	0.95
**S30**	13.8	0.5	0.90
**S80**	4.6	6.6	0.90
**Polydisperse NP**	2.6	0.2	0.69

**Table 3:** Pearson correlation analysis between the detected and the highly adsorbed proteins, for each physicochemical features and NP size (S10, S30, and S80). For each size, column contain: the Pearson product moment correlation coefficient (***ρ***); the related *p*-value adjusted for multiple testing using the Benjamini-Hochberg correction (*p*-value); the Bayes factor log odd-ratios (*log_e_*(*BF_01_*)) in favour of the null hypothesis. Protein features are respectively: the sequence length (*SeqLen*); the percentage of Arg AA (*%Arg*); the percentage of positively charged AA (*%PosAA*); the percentage of AA in disordered regions (*%DisReg*); the percentage of AA in hydrophobic clusters (*%HyClus*).

	S10			S30			S80		
	*ρ*	*p*-value	*log_e_(BF_01_)*	*ρ*	*p*-value	*log_e_(BF_01_)*	*ρ*	*p*-value	*log_e_(BF_01_)*
**SeqLen**	0.26	<2.2 × 10^−16^	−77.59	0.26	<2.2 × 10^−16^	−82.64	0.27	<2.2 × 10^−16^	−85.14
**%Arg**	0.20	<2.2 × 10^−16^	−44.05	0.20	<2.2 × 10^−16^	−43.82	0.20	<2.2 × 10^−16^	−48.40
**%PosAA**	0.14	3.2 × 10^−11^	−18.57	0.13	1.4 × 10^−10^	−17.09	0.13	4.8 × 10^−11^	−18.14
**%DisReg**	0.31	<2.2 × 10^−16^	−115.72	0.24	<2.2 × 10^−16^	−67.11	0.25	<2.2 × 10^−16^	−73.41
**%HyClus**	−0.23	<2.2 × 10^−16^	−62.35	−0.16	7.0 × 10^−16^	−29.08	−0.17	<2.2 × 10^−16^	−32.46

## Supplementary Materials

The following are available online at https://www.mdpi.com/2079-4991/10/2/240/s1, ShotgunData: An Excel file with the nanoLC-MS/MS shotgun processed data; Figure S1: ANOVA for each feature between HAP subsets; Table S1: Distributions comparison of HAP versus detected (shotgun) subsets; Table S2: Log Fold-Change linear models parameters estimation; Table S3: Bayesian regression and factor analysis; Table S4: Top-down/Bottom-up Analysis; Figure S2: Venn diagram of the non-adsorbed proteins on the three silica nanoparticles; Figure S3: GFP tagged proteins adsorption on silica microparticles and microfibers.
