# Supplementary material for: Protein Corona Composition of Silica Nanoparticles in Complex Media: Nanoparticle Size does not Matter
Source: Nanomaterials (Basel). 2020 Jan 29;10(2):240. doi: 10.3390/nano10020240 (PMC7075126; doi:10.3390/nano10020240)

Supplementary Materials

List of figures and tables

- **Figure S1**: ANOVA for each feature between HAP subsets
- **Table S1**: Distributions comparison of HAP *versus* detected (shotgun) subsets
- **Table S2**: Log Fold-Change linear models parameters estimation
- **Table S3**: Bayesian regression and factor analysis
- **Table S4**: Top-down/Bottom-up Analysis
- **Figure S2**: Venn diagram of the non-adsorbed proteins on the three silica nanoparticles
- **Figure S3**: GFP tagged proteins adsorption on silica microparticles and microfibers

Figure S1: ANOVA for each feature between HAP subsets

Welch's onx10-way ANOVA for each protein features of the highly adsorbed proteins (HAP) subsbets of each SiNPs (S10 in green, S30 in orange, and S80 in purple). Distributions of the protein features are depicted as violin plots, with the quartiles and the median indicated as a black square, and the average μ as a red dot (the average value is reported beside). The subtitle of each chart contains: the Fisher statistics (*F*) and the related p-value (*p*); the effect size (ω_p^2) and the 95% confidence interval for effect size estimate ($CI_{95\%}$ computed with 100 bootstrap samples); the total number of observations (n_{obs}). The caption below the chart contains the results of a Bayes factor analysis with the $\log_e$(BF) value (in favor of the null hypothesis) and the scale *r* of the prior Cauchy distribution (centered on zero) of effect sizes. These plots have been generated using the ggstatsplot R package (doi:10.5281/zenodo.2074621).

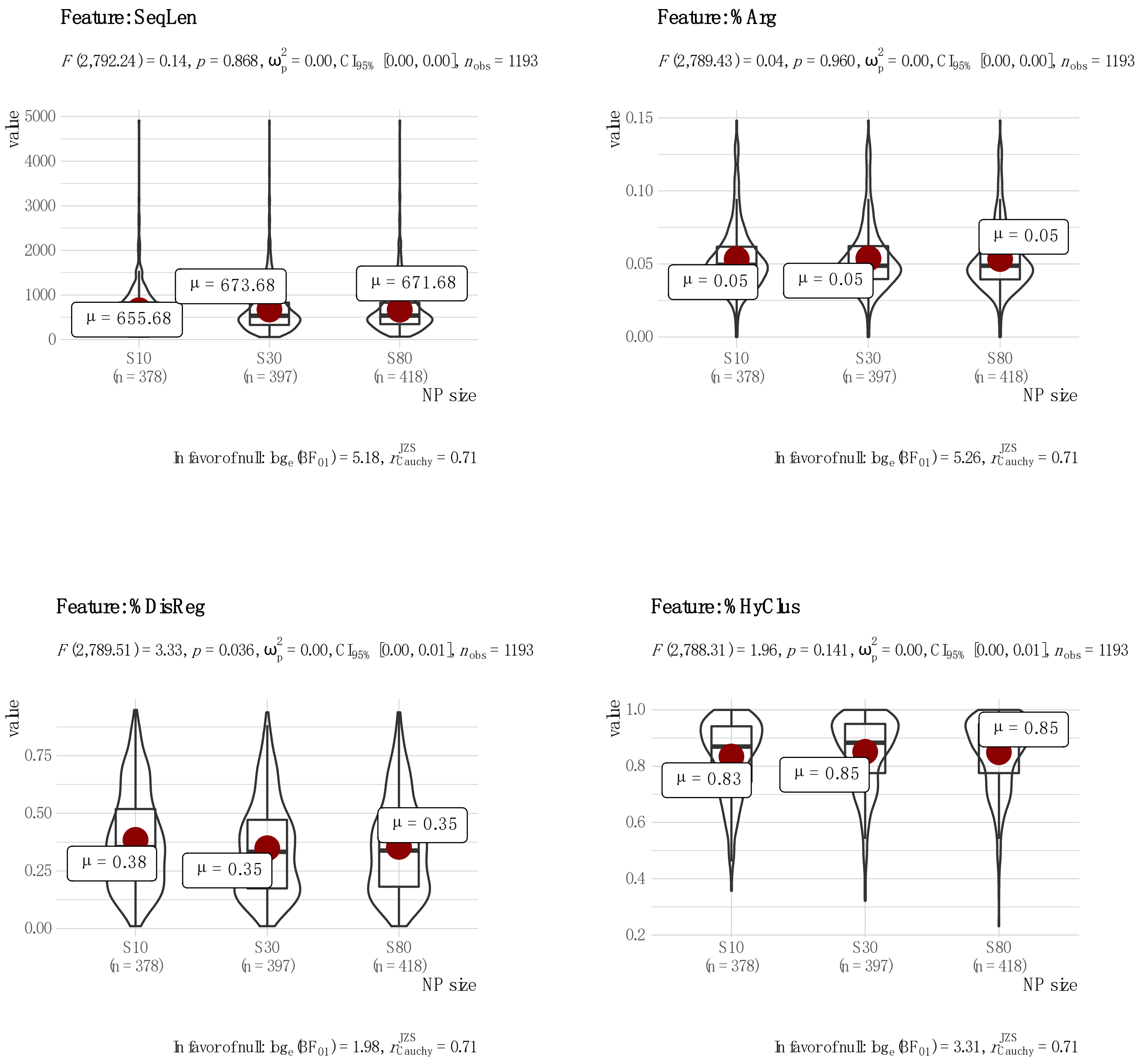

Feature: % PosAA

$F(2, 790.26) = 0.09$, $p = 0.912$, $\omega_p^2 = 0.00$, $CI_{95\%}$ [0.00, 0.00], $n_{obs} = 1193$

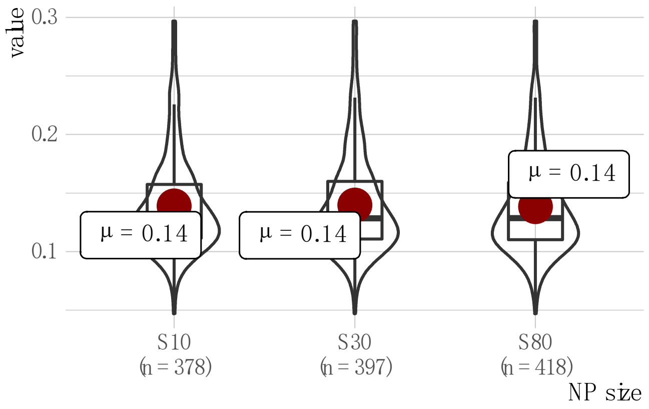

In favor of null: $\log_e(BF_{01}) = 5.21$, $r_{Cauchy}^{JZS} = 0.71$

Table S1: Distributions comparison of HAP *versus* detected (shotgun) subsets

Distributions of the HAP subset *vs* the whole detected proteins dataset have been compared using two statistical tests. The onx10-sided Kolmogorov-Smirnov test was used under the null hypothesis that the true cumulative distribution of HAP is above/below (as specified in the alternative column of the table) the cumulative distribution function of the detected set. Calculations were performed using R, excepted for the discrete "*Protein Length*" (SeqLen) features where a bootstrap version of the univariate Kolmogorov-Smirnov test (using 100 bootstraps) was used as implemented by the *ks.boot* function of the Matching R package. To compare the location of the distributions onx10-sided Wilcoxon rank sum test was also performed under the null hypothesis that the AP distribution is shifted to the right/left of the detected one. Calculations were performed using R. Tests p-values resulted from our calculations are reported in the tables.

Onx10-sided Kolmogorov-Smirnov Test

			p-values	
**Features**	**Alternative**	**S10**	**S30**	**S80**
**SeqLen** (Protein AA sequence length)	greater	0.733	0.660	0.752
**%Arg** (% of arginin residues)	greater	0.993	0.994	0.995
**%PosAA** (% of positively charged AA)	greater	0.999	0.999	0.999
**%DisReg** (% of disordered regions)	greater	0.991	0.977	0.989
**%HyClus** (% of hydrophobic clusters)	lesser	0.995	0.999	1.000

Onx10-sided Wilcoxon Rank Sum Test

			p-values	
**Features**	**Alternative**	**S10**	**S30**	**S80**
**SeqLen** (Protein AA sequence length)	greater	$4.3x10^{-05}$	$2.7x10^{-04}$	$1.0x10^{-04}$
**%Arg** (% of arginin residues)	greater	$8.7x10^{-12}$	$3.7x10^{-12}$	$5.0x10^{-12}$
**%PosAA** (% of positively charged AA)	greater	$2.8x10^{-11}$	$3.6x10^{-11}$	$1.6x10^{-10}$
**%DisReg** (% of disordered regions)	greater	$5.8x10^{-14}$	$3.0x10^{-07}$	$3.1x10^{-08}$
**%HyClus** (% of hydrophobic clusters)	lesser	$3.1x10^{-09}$	$6.4x10^{-05}$	$1.4x10^{-05}$

Table S2: Log Fold-Change linear models parameters estimation

Estimation of the linear regression parameters with the standard-error, and the associated F-test (statistics and p-value) for the three sizes of silica nanoparticles (S10, S30, and S80).

**S10**	**Estimate**	**Standard-error**	**F-test**	**p-value (F-test)**
β_0 (intercept)	-2.6559	0.1693	n/a	n/a
β_1 (SeqLen)	0.0019	0.0001	199.21	1.72×10^{-43}
β_2 (%DisReg)	3.4797	0.2679	168.77	2.52×10^{-37}
β_3 (%Arg)	24.5765	2.9272	70.49	7.85×10^{-17}
**S30**	**Estimate**	**Standard-error**	**F-test**	**p-value (F-test)**
β_0 (intercept)	-2.7636	0.1834	n/a	n/a
β_1 (SeqLen)	0.0021	0.0001	212.85	3.03×10^{-46}
β_2 (%DisReg)	2.6233	0.2957	78.71	1.38×10^{-18}
β_3 (%Arg)	29.1552	3.1845	83.82	1.13×10^{-19}
**S80**	**Estimate**	**Standard-error**	**F-test**	**p-value (F-test)**
β_0 (intercept)	-2.9929	0.1870	n/a	n/a
β_1 (SeqLen)	0.0021	0.0001	223.86	1.79×10^{-48}
β_2 (%DisReg)	2.7847	0.2987	86.93	2.45×10^{-20}
β_3 (%Arg)	31.2598	3.2528	92.35	1.74×10^{-21}

note: this analysis is limited to the parameters we deemed best to be involved in the adsorption process. These models exhibit a limited variance explained (adjusted R^2 index are 0.18, 0.15 and 0.16 for the NPs S10, S30, and S80 respectively), and have limited predictive power (which was beyond the scope of this study). Accuracy could be improved using various modelling techniques (*e.g.* using protein descriptors that account for the protein AA order, filtering the outliers, using nonlinear models), still the aim was not to explain LFC variations but to decipher the relationships between protein features driving the corona formation. Moreover, protein adsorption events are more complex and involved structural conformation modifications that cannot be tackled using only the primary sequence of the proteins.

Table S3: Bayesian regression and factor analysis

For each size of SiNP, the best linear regression model, using the log fold-change as a response variable and all physicochemical properties as covariates, has been selected with the BayesFactor R package. The table summarises the top five regressions models and the probability ratio against the best model (indicated on the first line of the table). The first column contains the model covariates, added to the best model (in blue), discarded from the best model (in orange).

S10

Model covariates	Probability ratio (*vs* the first/best model)
SeqLen + %DisReg + %Arg	1.000
SeqLen + %DisReg + %Arg + %PosAA	0.964
SeqLen + %DisReg + %Arg + %HyClus	0.116
SeqLen + %DisReg + %Arg + %PosAA + %HyClus	0.096
SeqLen + %DisReg ~~+ %Arg~~ + %PosAA	5.060×10^{-13}

S30

Model covariates	Probability ratio (*vs* the first/best model)
SeqLen + %DisReg + %Arg	1.000
SeqLen + %DisReg + %Arg + %PosAA	0.182
SeqLen + %DisReg + %Arg + %HyClus	0.070
SeqLen + %DisReg + %Arg + %PosAA + %HyClus	0.015
SeqLen ~~+ %DisReg~~ + %Arg + %HyClus	2.356×10^{-08}

S80

Model covariates	Probability ratio (*vs* the first/best model)
SeqLen + %DisReg + %Arg	1.000
SeqLen + %DisReg + %Arg + %PosAA	0.328
SeqLen + %DisReg + %Arg + %HyClus	0.069
SeqLen + %DisReg + %Arg + %PosAA + %HyClus	0.025
SeqLen ~~+ %DisReg~~ + %Arg + %HyClus	8.496×10^{-09}

Table S4: Top-down/Bottom-up Analysis

We have also performed top-down/bottom-up analysis wherein the former each independent variable is eliminated/added one at a time. Tables *a-c* contain the top-down analysis result, where each covariate is eliminated, one at a time, from the full model (*i.e.* with all covariates). The change in Bayes Factor (BF) is reported, against the full model. For example, a change in BF of 10 means that a model without the omitted covariate is 10 times more probable than the full model. Conversely, a change of 10^{-01} or 1/10 is ten times less probable than the full model. Tables *d-f* contain the bottom-up analysis, where each covariate is added, one at a time, to the intercept model (*i.e.* the model with one covariate is compared to a constant value model). Similarly to the top-down analysis, the change of Bayes factor is reported in the second column. For example, a value of 10 means that the model with only this covariate is ten times more probable than a constant model.

a) S10 — Top-Down Analysis

Model covariates	Change in BF
Omit %HyClus	11.090
Omit %PosAA	1.214
Omit %Arg	6.100×10^{-13}
Omit %DisReg	3.728×10^{-14}
Omit SeqLen	3.850×10^{-38}

b) S30 — Top-Down Analysis

Model covariates	Change in BF
Omit %HyClus	12.390
Omit %PosAA	4.765
Omit %Arg	4.538×10^{-13}
Omit %DisReg	1.341×10^{-07}
Omit SeqLen	4.460×10^{-40}

c) S80 — Top-Down Analysis

Model covariates	Change in BF
Omit %HyClus	12.970
Omit %PosAA	2.729
Omit %Arg	4.170×10^{-15}
Omit %DisReg	2.428×10^{-08}
Omit SeqLen	5.252×10^{-43}

d) S10 — Bottom-Up Analysis

Model covariates	Change in BF
%HyClus	1.342×10^{27}
%PosAA	1.524×10^{08}
%Arg	1.607×10^{19}
%DisReg	1.745×10^{50}
SeqLen	5.279×10^{33}

e) S30 — Bottom-Up Analysis

Model covariates	Change in BF
%HyClus	5.352×10^{12}
%PosAA	3.507×10^{07}
%Arg	1.283×10^{19}
%DisReg	1.545×10^{29}
SeqLen	8.173×10^{35}

f) S80 — Bottom-Up Analysis

Model covariates	Change in BF
%HyClus	1.568×10^{14}
%PosAA	9.948×10^{07}
%Arg	1.236×10^{21}
%DisReg	8.276×10^{31}
SeqLen	9.964×10^{36}

Figure S2: Venn diagram of the non-adsorbed proteins on the three silica nanoparticles

This diagram depicts the number of shared non-adsorbed proteins, in all possible overlapping sets, on the three silica nanoparticles S10, S30, and S80. Non-adsorbed proteins are defined with the same method used to select the HAP (see Material and Methods), but with a Log Fold-Change threshold of ≤-1 instead of ≥1.

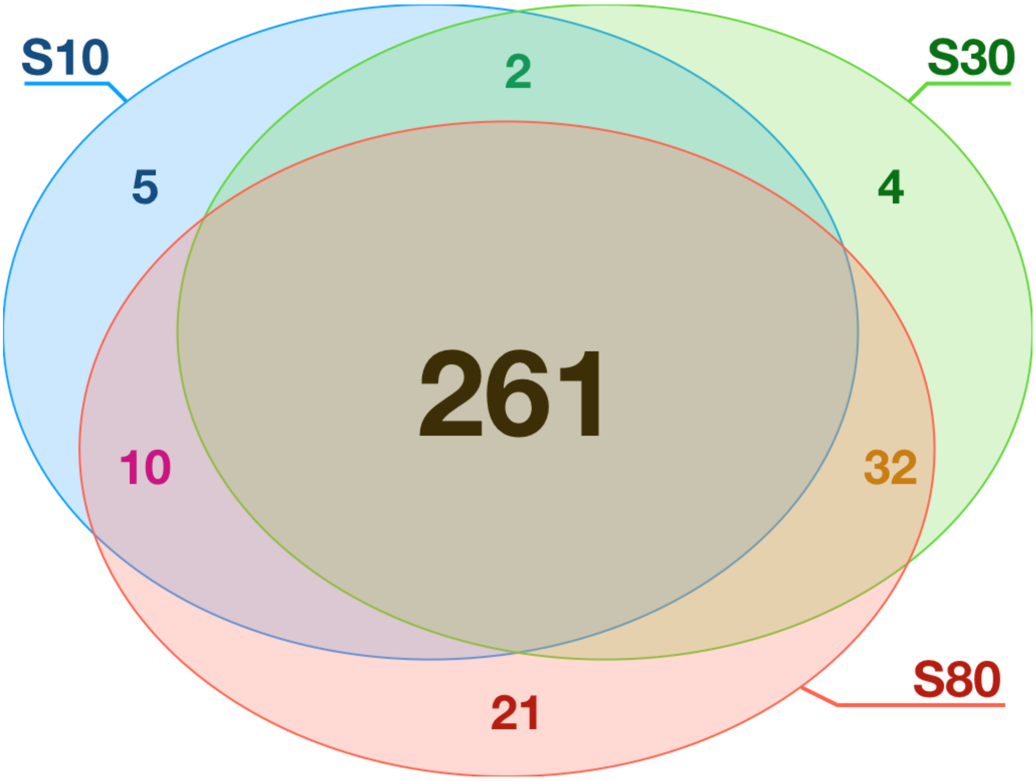

Figure S3: GFP tagged proteins adsorption on silica microparticles and microfibers

Images were obtained using GFP tagged yeast proteins and quartz microparticles (purchased from Sigma-Aldrich) or microfibers (made by Whatman). Panels A-D are representative images obtained by epifluorescence microscopy (A-C) or bright field microscopy (D). A) GFP-Rpl35b chimeric protein with microparticles (median Log_2 Fold-Change = 1.13 with S10, S30, and S80 SiNPs); B) GFP-Tef1 chimeric protein with microfibers (median Log_2 Fold-Change = 1.33); C) GFP-Pgi1 chimeric protein with microparticles (median Log_2 Fold-Change = -4.29); D) GFP-Pgi1 chimeric protein with microparticles (bright field). The same image corrections have been applied to the three images A,B, and C for direct comparison of fluorescence levels. Scales bars, depicted as white or black rectangles at the bottom right of each panel, represent 20µm of length.

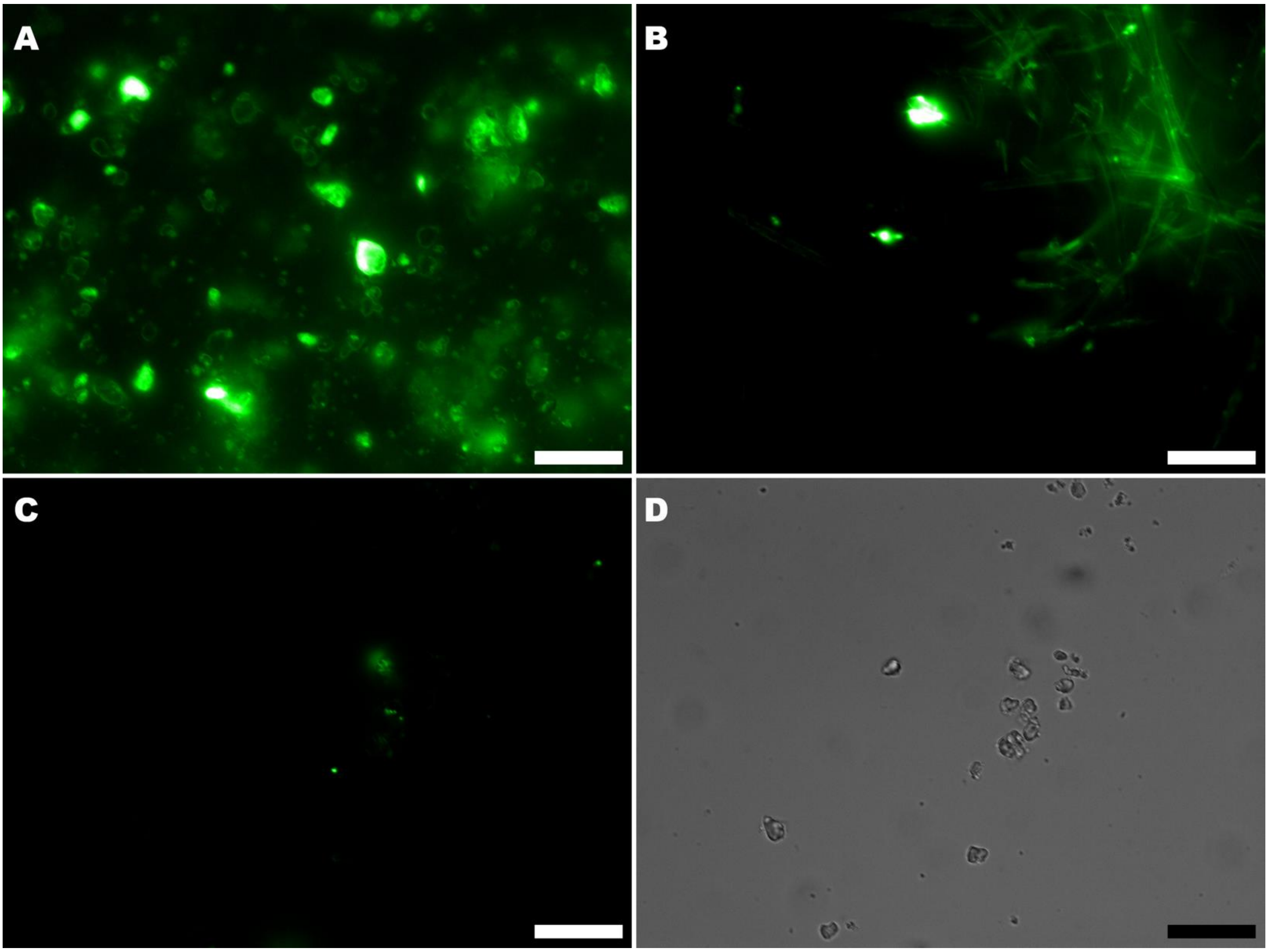

Supplement: Supplementary file 1 [file nanomaterials-10-00240-s001.zip › nanomaterials-685518-supp/Supplementary materials.pdf]
